# Attention mediates the influence of numerical magnitude on temporal processing

**DOI:** 10.1038/s41598-021-90466-2

**Published:** 2021-05-26

**Authors:** Anuj Shukla, Raju S. Bapi

**Affiliations:** Cognitive Science Lab, Kohli Research Centre on Intelligent Systems, International Institute of Information Technology, Hyderabad, India

**Keywords:** Psychology, Human behaviour

## Abstract

The processing of time and numbers has been fundamental to human cognition. One of the prominent theories of magnitude processing, *a theory of magnitude* (ATOM), suggests that a generalized magnitude system processes space, time, and numbers; thereby, the magnitude dimensions could potentially interact with one another. However, more recent studies have found support for domain-specific magnitude processing and argued that the magnitudes related to time and number are processed through distinct mechanisms. Such mixed findings have raised questions about whether these magnitudes are processed independently or share a common processing mechanism. In the present study, we examine the influence of numerical magnitude on temporal processing. To investigate, we conducted two experiments using a temporal comparison task, wherein we presented positive and negative numerical magnitudes (large and small) in a blocked (Experiment-1) and intermixed manner (Experiment-2). Results from experiment-1 suggest that numerical magnitude affects temporal processing only in positive numbers but not for negative numbers. Further, results from experiment-2 indicate that the polarity (positive and negative) of the numbers influences temporal processing instead of the numerical magnitude itself. Overall, the current study seems to suggest that cross-domain interaction of magnitudes arises from attentional mechanisms and may not need to posit a common magnitude processing system.

## Introduction

Over the last two decades, numerous studies have advanced our knowledge of how humans utilize perceptual information to estimate magnitudes such as space, time, and number. One of the most popular theories of magnitude processing, *a theory of magnitude* (ATOM), suggests that a generalized magnitude system^1^ processes information related to space, time, and numbers and converts these into a common currency. Growing evidence from neuroimaging studies has supported the idea of common magnitude processing and reported that cross-domain magnitude interaction occurs in the prefrontal and parietal cortices in the brain^2–5^. On a behavioral level, many studies have found support in favor of a shared magnitude system^6–8^ and argued that cross-domain magnitude interactions arise because magnitudes dimensions are processed through a generalized system. On the contrary, many researchers have provided evidence against the existence of such a generalized magnitude system^9–14^. Such mixed evidence has raised a question on the nature of the cross-domain magnitude interaction and its neuro-cognitive mechanisms. Suppose we assume that the common metric system does not process space, time, and numbers. In that case, we need to provide an alternative explanation for the results from the existing literature that found evidence for ATOM. In the present paper, we investigated whether the influence of one magnitude on the processing of the other magnitude results from a common processing mechanism or is mediated by cognitive factors. Specifically, here we examine the influence of task-irrelevant numerical magnitude on temporal perception.

Studies investigating temporal perception have shown that perceived duration is often influenced by emotion^15^, temporal frequencies^16^, motion^17,18^, color^19,20^, and resulted in distortions in temporal judgments for certain types of stimuli over others (for example, blue colored stimuli are perceived to last longer than red colored^20^). Apart from these stimulus features, researchers have demonstrated that temporal processing of stimuli is also affected by task-irrelevant, non-temporal stimulus properties such as stimulus magnitude (number and size)^21,22^. It has been well documented that a large-magnitude stimulus is judged to last longer than a small-magnitude stimulus. Specifically, in the context of duration judgments, the duration of a visual stimulus that is more bright, more numerous, or larger in size is perceived to last longer when participants were asked to select the stimulus that lasted longer in time^21–23^. Similarly, in the temporal reproduction task, participants reproduced longer durations for numbers that are large in magnitude compared to numbers that are small in magnitude^24–27^. Such cross-domain monotonic mapping (large = longer and small = shorter) has been reported across different contexts^28^ and timescales^29–31^. The proponents of the generalized magnitude system have suggested that such cross-domain monotonic mapping arises from a common magnitude processing system, as proposed by ATOM. Conversely, others have provided convincing evidence against such common magnitude processing and argued in favor of domain-specific processing. For example, in a recent study, participants were tested on numerosity and temporal judgments in a dual-task setting. They were asked to remember letters while making numerical or temporal judgments. Authors hypothesized that if a common magnitude processing were operating, both numerosity and temporal processing would show similar biases under the cognitive load condition. However, the result pointed out that cognitive load led to differential biases across two magnitude domains. Specifically, under cognitive load conditions, participants underestimated numerosity in the numerosity task, whereas they overestimated duration in the temporal judgment task^32^. Similar results have also been noted when participants were asked to make judgments about time and numerosity under the influence of emotion^33^. Such differential bias across different magnitude processing streams indicates that numbers and time are processed through domain-specific magnitude processing systems rather than being processed by a generalized magnitude system.

Further, it has also been argued that if space, time, and numbers are processed through a common code, then they would influence one another symmetrically. For example, the way number affects the processing of time (large number makes durations appear longer), time (or temporal duration) should also affect number processing (numerosity stimulus presented for longer duration appears to be more numerous).

Researchers have conducted a series of experiments using a Stroop task paradigm to investigate the bidirectional influence between number and time. Participants were presented with two magnitude dimensions, namely, size and numerosity, in a congruent and incongruent fashion. They were required to make judgments either for size or for numerosity. For the numerosity judgments, response time was observed shorter when size and numerosity were presented in a congruent manner than incongruent one^34^. On the other hand, numerosity did not affect the performance on the size (spatial) judgment task. In another study^35^, participants had to perform spatial and temporal reproduction tasks. They were presented with either lines or dots on the computer screen and were required to reproduce the duration or spatial displacement on different trials. Overestimation of duration was noted for longer lines and underestimation of duration for shorter lines. However, no effect of duration was observed on the spatial reproduction task. It has been observed that the presentation of varying word lengths influenced duration judgments. Specifically, the estimation of duration increases as a function of the length of the word^36^. For example, the word “train” was perceived to last longer on the screen than the word “pen”. However, spatial judgments about word length were not affected by the duration. This indicates a lack of bidirectional influence in magnitude interactions. Similar asymmetries have also been observed for time and number interaction. In an investigation of time and number, it was observed that number magnitude information affected temporal processing but not vice-versa. In this study, a Stroop task paradigm was used to present congruent and incongruent conditions incorporating numerical and duration information. In the duration judgment task, participants were faster when the number and duration information was presented in a congruent order compared to incongruent conditions. Like previous studies, no effect of duration was observed for the numerosity judgment task^37^.

The interaction between numerosity and time has also been studied using an adaptation paradigm^14^. It is assumed that if number and time are processed through the common magnitude system, adaptation to numerosity would affect the processing of duration, and adaptation to duration would interfere with numerosity processing. Interestingly, the authors observed a unidirectional effect of adaptation to the duration on numerosity but not the other way around. A more recent study investigated the influence of implicit and explicit processing of duration on the number and vice-versa. Participants were relatively faster and more accurate in explicit processing conditions when the large numerical magnitude was paired with long duration than when paired with short durations. On the contrary, no facilitation was observed when the small numerical magnitude was presented with short durations. Further, participants' accuracy for long-duration judgments was relatively better after processing large numerical magnitudes compared to that after processing small numerical magnitudes. Interestingly, an opposite pattern was observed for short durations^38^. Researchers have argued that numerical information affects temporal processing more than the other way around.

Apart from the above studies, a handful of studies have suggested the role of visuospatial attention in such cross-domain magnitude interaction. To understand the role of visuospatial attention, number and time magnitude were presented in the left and right visual space. Authors reported temporal overestimation on the right and underestimation in the left visual space, independent of the numerical magnitude. However, numerical magnitude biased temporal estimation only when the numbers were presented at the center^39^. We have recently shown that numerical magnitude selectively affects the accuracy but not the precision of temporal judgments and argued that such selective bias might result from visuospatial attention^40^.

These conflicting findings raise a question as to whether the magnitude information of time and number domains is processed independently or shares a common processing mechanism. This remains an open and highly debated subject.

In the present study, we investigate whether the numerical magnitude interferes with temporal processing using positive and negative numbers. The *negative number* is an interesting case. Unlike a positive number, the relation between the numerical magnitude to the absolute value of a number is the opposite in the case of a negative number. For example, − 1 is bigger than − 9 when considering the absolute values, but 1 is smaller than 9. Thus, from a magnitude perspective, − 1 is larger than − 9, whereas, in the case of positive numbers, this relation is reversed; one (1) is smaller in number and magnitude as compared to nine (9).

It has been shown that merely perceiving positive numbers can cause an automatic shift of attention in the mental space (such as on a *mental number line*) without making an eye-movement^41^. However, negative numbers may not be effective in inducing such shift of spatial attention^42^. Given the differences in the processing or the cognitive representation of negative numbers, it would be interesting to understand how negative numerical magnitudes interact with duration judgments. Such a study would also be particularly interesting to understand whether numerical magnitude affects/interferes with temporal judgments directly or is mediated by a shift of attention evoked due to the numbers.

To examine the influence of numerical magnitude on temporal processing, we conducted two experiments using a temporal comparison task. We presented positive and negative numerical magnitudes in a blocked (Experiment-1) and intermixed manner (Experiment-2). We hypothesized that if a generalized magnitude system processes the magnitudes of both time and number, we would expect numerical magnitude to influence duration judgments independent of the number format (positive and/or negative) in both the experiments.

## Results

### Experiment-1: positive and negative number magnitudes (block design)

All the subjects performed a *Positive* and *Negative number block*. On each trial, subjects performed a duration discrimination task for the two sequentially presented number magnitudes. In the positive number block, the first magnitude (standard) was “5” and had a fixed duration of 500 ms. The second magnitude (comparison) was either “1” or “9” presented with varying durations from 100 to 900 ms in steps of 100 ms. Similarly, in the negative number block, everything was presented like in the positive block, but the number was presented with a—(negative) sign. The standard- and comparison-stimuli were separated by an inter-stimulus-interval (ISI). Subjects were instructed to judge whether the duration that the comparison stimulus lasted was longer or shorter compared to that of the standard stimulus.

**Figure 1:**
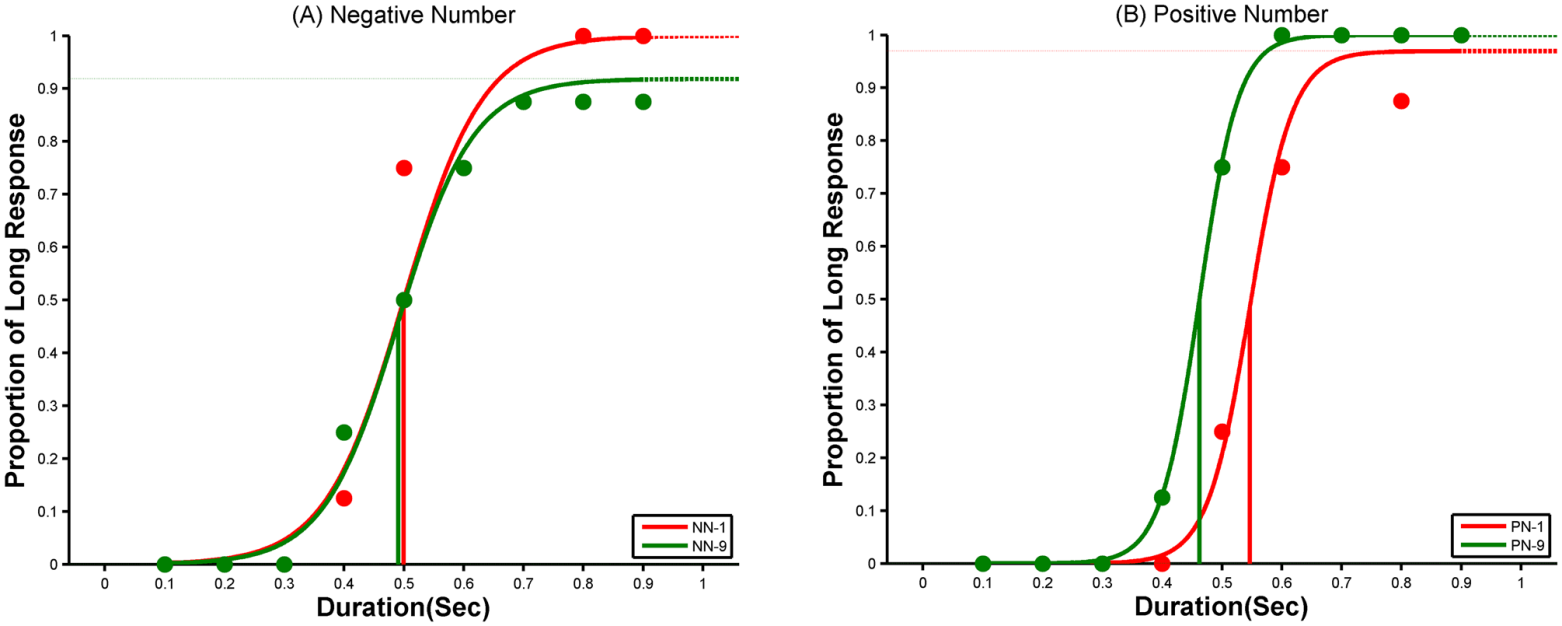
Psychometric fit for the results of a representative participant (Experiment-1). The left panel shows a psychometric plot of small and large numerical magnitudes for negative number (NN) blocks, and the right panel shows the same for the positive number (PN) blocks.

The responses were recorded in terms of “long” and “short” keypresses on the keyboard. 35 participants took part in the experiments. However, two participants could not complete one of the experimental blocks, and another two participants’ data failed to fit the psychometric function in one of the magnitude conditions. Therefore, their data were excluded from the final analysis. Hence, the statistical analysis reported here includes data from 31 participants. We calculated the *point of subjective equality* (PSE)—the duration at which 50% of the time comparison stimulus duration was judged longer compared to the standard stimulus duration. Lower the PSE value higher the overestimation of time, and vice-versa. The PSE was estimated using *Psignifit4 (a MATLAB-based toolbox)* by fitting a *Logistic Function* to each numerical magnitude (small, large) data across positive and negative blocks (see Fig. 1). Thus, a total of four PSEs were estimated for each participant. The average PSE plots for both the positive and negative blocks are included in the supplementary material.

In the positive number block, the PSE for large magnitude, i.e., for “9,” was 469.80 ± 68.35 ms (mean ± SD), whereas the PSE for the small magnitude, i.e., for “1” was 506.21 ± 57.04 ms. Similarly, in the negative number blocks, the PSE for large magnitude, i.e., for “ − 1” was 491.14 ± 66.63 ms, whereas the PSE for small magnitude “ − 9” was 472.53 ± 78.14 ms.

**Figure 2:**
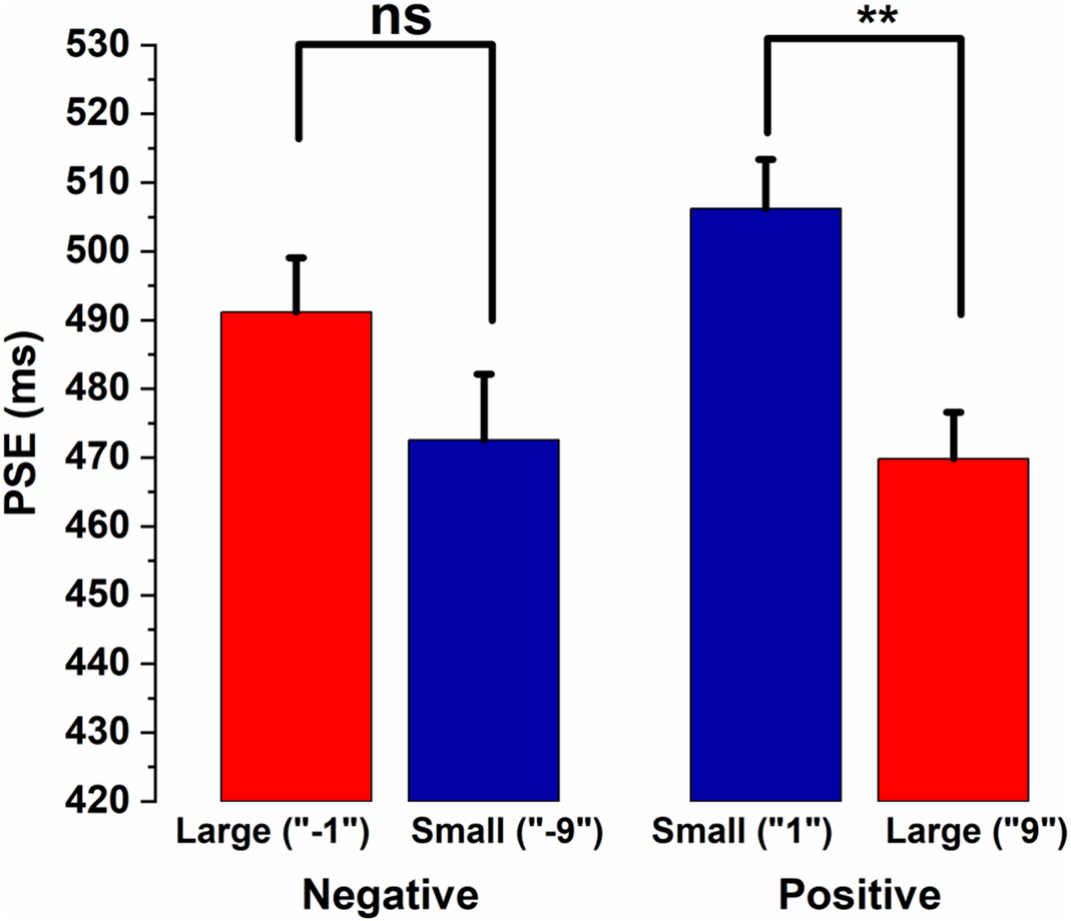
Comparison of PSE values across positive and negative number blocks (Experiment-1). Average PSE values of small and large numerical magnitude trials for positive and negative number blocks. The error bar indicates standard error of the mean, estimated using a method appropriate for within-subject designs^43,44^. **Indicates statistically significant differences (*p* < 0.01).

To test whether numerical magnitudes interfere with temporal processing, we used 2 (Block: Positive vs Negative) × 2 (Magnitude: Small vs Large) repeated measures ANOVA. The results suggest that the main effect of blocks was not significant, *F*(1, 30) = 0.635, *p* = 0.432, partial η^2^ = 0.021. Similarly, the main effect of numerical magnitudes was also non-significant, *F*(1, 30) = 0.877, *p* = 0.356, partial η^2^ = 0.028. However, the Block × Magnitude interaction was found to be significant, *F*(1, 30) = 18.780, *p* = 0.001, partial η^2^ = 0.385. Further, the simple main effects analysis (see Fig. 2) indicates that the numerical magnitude affects temporal processing in positive number blocks, *F*(1, 30) = 15.128, *p* = 0.001, partial η^2^ = 0.335 but not in the negative number blocks: *F*(1, 30) = 1.995, *p* = 0.168, partial η^2^ = 0.062. The null result observed for the negative number block has also been investigated using the Bayesian approach (see supplementary materials).

At the end of the experiment, participants were given a debrief task wherein the numbers (positive or negative) were presented side by side. The order of the presentation of numbers was counterbalanced across participants. Participants were asked to indicate which of the numbers was larger in magnitude. All the participants indicated the large magnitude number in both positive and negative number comparisons with 100% accuracy. This indicates that the sense of numerical magnitude (large and small) remained intact in both positive and negative number comparisons.

### Experiment-2: positive and negative number magnitudes (intermixed design)

Like in experiment-1, subjects performed the duration discrimination task. In this experiment, we presented the positive and negative numbers in an intermixed order. Unlike in experiment-1, the first magnitude (standard) was “0” and had a fixed duration of 500 ms. The second magnitude (comparison) was either “1”, “9”, “ − 1”, or “ − 9” presented with varying durations from 100 to 900 ms in steps of 100 ms. The standard and the comparison stimuli were separated by an inter-stimulus-interval (ISI). Participants were instructed to judge whether the duration of the comparison stimulus lasted longer or shorter than the standard stimulus.

**Figure 3:**
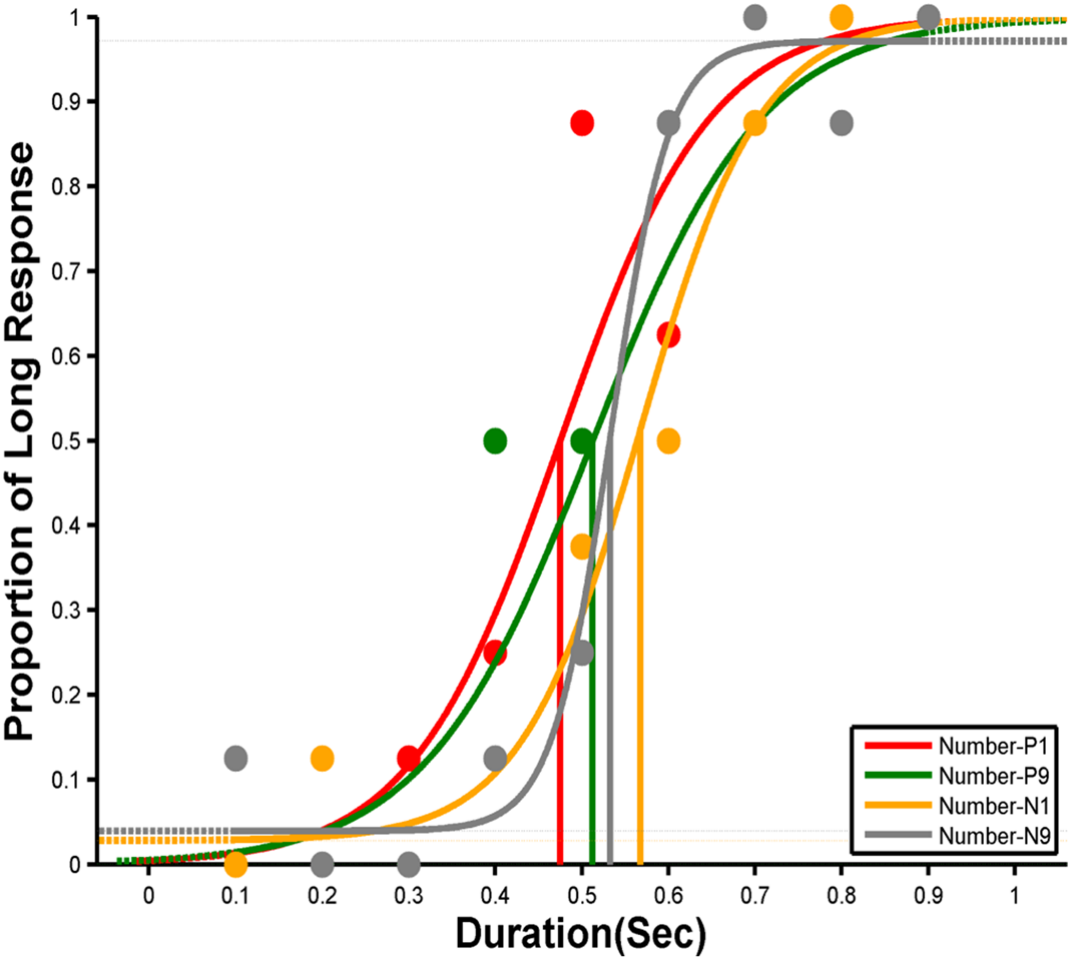
Example psychometric fit for a representative participant (Experiment-2). In the positive number domain, the small magnitude “1” (Number-P1) is shown in red color, and the large numerical magnitude “9” (Number-P9) is presented in green color. In the negative number domain, the small numerical magnitude “ − 9” (Number-N9) is indicated in gray color, and the large numerical magnitude “ − 1” (Number-N1) in yellow color.

Data was collected from 31 participants. However, one participant could not complete the task, and another participant’s data did not fit the psychometric function in one of the magnitude conditions. Therefore, their data were excluded from the final analysis. Hence, the statistical analysis reported here includes 29 participants’ data. Like in experiment-1, PSE was estimated using *Psignifit4 (MATLAB-based toolbox)* by fitting a *Logistic Function* to each numerical magnitude (small, large) data for both positive and negative numbers (see Fig. 3). Thus, a total of four PSEs were estimated for each participant in experiment-2 as well. The average PSE plots are included in the supplementary material.

A one-way repeated measures ANOVA was used to compare whether the temporal processing for the numerical magnitude (small and large) differs across positive and negative numbers. P*ost hoc* analysis (using the *Holm* correction to adjust *p*) was performed to probe the temporal processing differences across different numbers.

**Figure 4:**
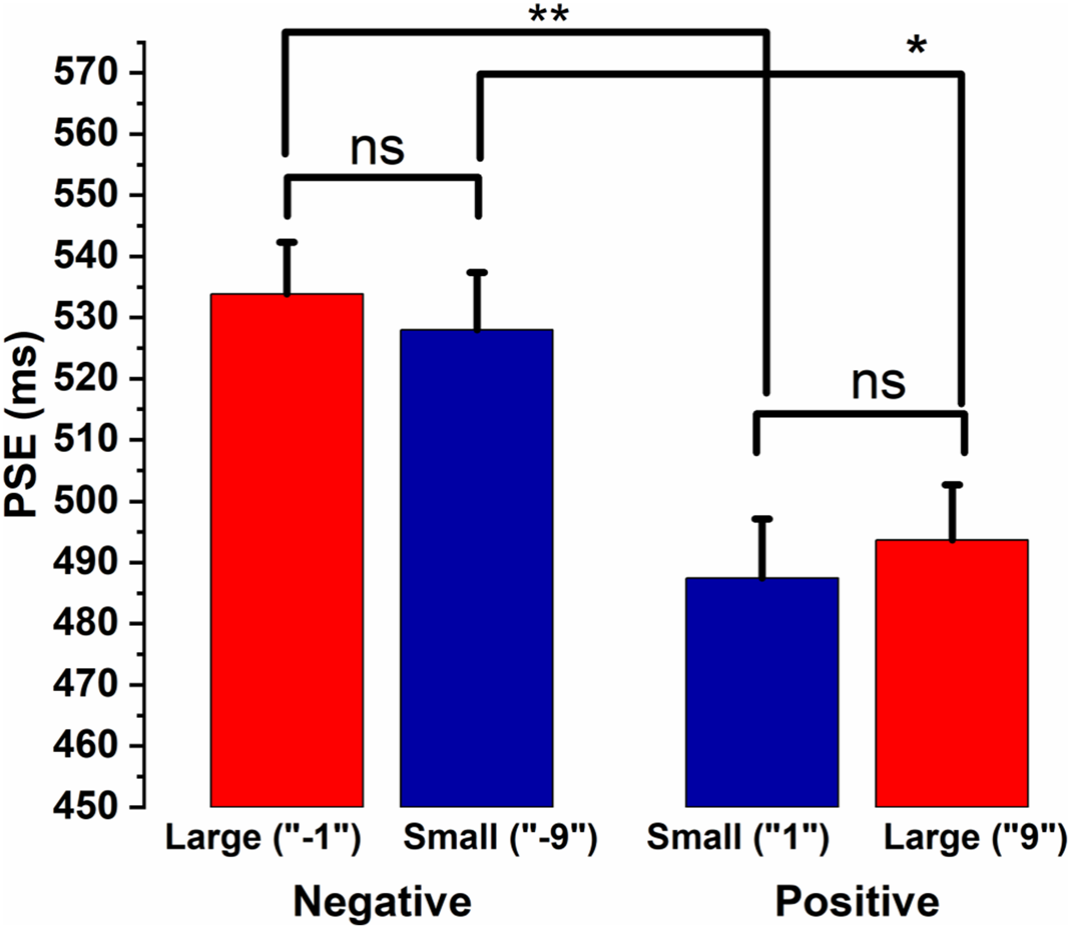
Comparison of PSE values across different numbers (Experiment-2). Average PSE values of small and large numerical magnitudes are shown for positive and negative numbers. The error bar indicates standard error of the mean, estimated using a method appropriate for within-subject designs^43,44^. *Indicates statistically significant differences (*p* < 0.05) and **represents (*p* < 0.01).

The ANOVA results (see Fig. 4) revealed that the PSEs were significantly different from one another *F*(3, 84) = 6.592, *p* = 0.001, partial η^2^ = 0.191. The post hoc analysis (using the *Holm* correction to adjust *p*) suggests that the duration for “1” was significantly overestimated compared to “ − 1” (*p* = 0.004). Similarly, the duration for “9” was also significantly overestimated compared to “ − 9” (*p* = 0.029). Further, the duration of large positive numerical magnitude (“9”) was significantly overestimated than that of the large negative magnitude (“ − 1”) (*p* = 0.012). Similarly, overestimation of duration was also observed for the small positive numerical magnitude (“1”) compared to the small negative magnitude (“ − 9”) (*p* = 0.012). However, we observed no significant differences in the PSEs when compared within the positive and negative numerical magnitudes (1 versus 9 and − 1 versus − 9). Further, the Bayesian approach was used to estimate the magnitude of the null results (see supplementary materials).

In addition to the above ANOVA, we analyzed whether the temporal perception is different for the positive and negative numbers in general. To test this, we computed the average PSEs for positive (1 and 9) and negative (− 1 and − 9) numbers and submitted them to a paired samples t-test. The results suggest that the duration for the positive numbers was significantly overestimated (490.57 ± 67.39) compared to that of the negative numbers (530.91 ± 67.69), t(28) = 3.795, *p* = 0.001, Cohen’s *d* = 0.70.

Like in experiment-1, participants' knowledge about the magnitude (large and small) was tested by presenting the two numbers side by side and asking them to indicate which number was larger in terms of magnitude. All the participants indicated the large magnitudes with 100% accuracy for both positive and negative number domains. This suggests that the participants were aware of large and small numerical magnitudes in positive and negative number cases.

## Discussion

In the current study, we investigated the influence of positive and negative numerical magnitudes on duration judgments. We proposed that larger numerical magnitude would be perceived to last longer in time than smaller numerical magnitude irrespective of the number format in line with ATOM and previous studies. We used a temporal-discrimination task to present positive and negative numbers. We estimated the temporal accuracy in terms of point of subjective equality (PSE)—the point at which participants judged the comparison duration to be longer or shorter with 50% accuracy for each numerical magnitude (small and large) and number domain (positive and negative).

Previous studies have shown that task-irrelevant numerical magnitude can interfere with temporal processing^21,22,24,25^. For example, durations tend to be overestimated in the presence of large numerical magnitude and underestimated with small numerical magnitudes. Such interference has been attributed to common magnitude processing proposed by ATOM^1^. Our results indicate that numerical magnitude affects temporal processing only in the case of positive numbers but not in the case of negative numbers (Experiment-1). When “ − 1” and “ − 9” were presented in the same block (negative number block) along with the standard duration presented in conjunction with “ − 5”, the results indicated that numerical magnitude did not interfere with temporal processing. However, in the positive number block, we observed an overestimation of time for the large numerical magnitude (“9”) compared to the small numerical magnitude (“1”). Thus, our results from experiment-1 suggest that magnitude affects temporal processing only when presented in a positive number format but not in a negative format. This result is particularly interesting considering that the participants had a clear sense of the relative magnitudes both in the positive and negative number domains as assessed during the debrief session. We suggest that the positive and negative blocks may be different due to the more frequent use of positive numbers in real life. Thus, the sense of magnitude in positive numbers seems intuitive as compared to negative numbers. Consequently, intuitive cross-domain magnitude mapping was feasible in positive number blocks but not in negative number blocks. It is also important to note that in the present study, participants were asked to make temporal judgments independent of the presented numerical magnitudes. Despite this, automatic processing of positive numbers seems to have affected duration judgment.

In experiment-2, four numbers (1, 9, − 1, and − 9) were presented against a common reference, “0”. In line with ATOM’s prediction, we expected two kinds of results. Firstly, differential temporal processing across positive and negative numbers, and secondly, within each specific number domain (positive, negative), participants would overestimate the durations in the presence of larger numerical magnitudes as compared to trials with smaller numerical magnitude. In particular, the second result related to the specific number domains is important to establish the generality of ATOM-like phenomenon. While assessing the temporal processing for positive and negative numbers, we observed overestimation of duration for positive numbers (“1” or “9”) compared to negative numbers (“ − 1” or “ − 9”). Results also indicate that large positive numerical magnitude (“9”) was perceived to last longer compared to large numerical magnitude (“ − 1”) in the negative domain. Similarly, overestimation of duration was observed for small positive numerical magnitude (“1”) compared to that of the small numerical magnitude of the negative number domain (“ − 9”). Interestingly, the temporal perception for the pairs of numbers that are equal in magnitude in the absolute sense (“1” vs “ − 1” and “9” vs “ − 9”, note here that |1| =|− 1| and similarly |9| =|− 9|) was also found to be different. Once again, duration overestimation takes place for positive numbers, but underestimation occurred for negative numbers. Therefore, our present results suggest that the duration of positive numbers would always be overestimated than that of negative numbers. Given the fact that positive numbers are large in magnitude compared to negative numbers, at first, this result seems consistent with the previous findings advocating ATOM’s proposal, i.e., overestimation in the presence of large numerical magnitudes and underestimation in the presence of small numerical magnitudes. On the contrary, ATOM’s prediction did not hold true when we looked at how large and small numerical magnitudes affected temporal processing within each number domain separately. Surprisingly, no temporal processing differences were observed when small and large numerical magnitudes were compared within the positive (“1” vs “9”) and negative (“ − 9” vs “ − 1”) number domains individually. Therefore, the present results are inconsistent with ATOM’s proposal and seem to indicate that temporal processing is not always affected by the presence of (task-irrelevant) numerical magnitudes.

The overall results from experiment-2 raise a fundamental question as to what kind of information is crucial in cross-domain magnitude processing/interaction. What would lead to overestimation/underestimation of duration? Is it the numerical magnitude (large or small) or the number format (positive or negative polarity) that result in these effects?

Findings from experiment-2 (see Fig. 4) indicate that positive numerical magnitudes (“1” or “9”) are perceived longer compared to negative numerical magnitudes (“ − 1” or “ − 9”). One way to analyze these results is in terms of magnitude. Since positive numbers are large in magnitude compared to negative numbers, one can argue that the results observed in the present study may be driven by the interference from a common magnitude processing system as posited by ATOM. However, when we examine the magnitude effects within each number domain (positive and negative, separately), the effects vanished, i.e., 1 and 9 as well as − 1 and − 9 are not perceived differently from the perspective of duration judgment. Such inconsistent results may indicate that the differential temporal processing of positive and negative numbers is modulated by number's polarity (positive and negative) but not by magnitudes per se. Further, suppose the numerical magnitude and durations were processed through a common magnitude system. In that case, we should have observed the influence of numerical magnitudes on temporal processing for positive and negative numbers. Yet, temporal perception of large and small numerical magnitudes within positive and negative number domains did not differ, providing further evidence in favor of processing of the polarity of the number. Hence, the results of experiment-2 should be attributed to the automatic processing of the sign (positive and negative) rather than magnitudes.

Taken together, the results of experiments 1 and 2 suggest that automaticity plays a crucial role in both the experiments. In experiment-1, positive numbers (1 and 9) have shown differential temporal processing but not negative numbers (− 1 and − 9). It could be that the relation between the numerical magnitude to the absolute value of a number becomes critical. The number-to-magnitude relation is different for positive and negative numbers. For example, − 1 is bigger than − 9. However, when considering the absolute values, 1 is smaller than 9. Thus, from a magnitude viewpoint, − 1 is larger than − 9, whereas, in positive numbers, this relation is reversed; one (1) is smaller in number and magnitude than nine (9). Therefore, we propose that such conceptual mapping (absolute number-to-magnitude) may evoke a sense of automaticity that leads to differential processing for positive and negative number blocks in experiment-1. Similarly, in experiment-2, the sense of automaticity is elicited by the numbers' polarity, leading to differential temporal processing for positive and negative numbers when presented in the same block against a common reference.

Alternatively, it has been argued that number and time are strongly associated with space and can be represented in the form of a mental number/timeline. Large magnitudes (number and time) are associated with the right side of space, and small magnitudes (number and time) are linked to the left side of space. Evidence from the number processing studies shows that the mere presence of numbers (small or large) induces shifts of attention (leftward or rightward, respectively) in the mental space^41^. Thus, it may be the case that automatic processing of a positive number influences temporal processing while causing a shift of attention in the mental space. Therefore, we suggest that the influence of positive numbers on temporal processing may be mediated by the spatial attention evoked due to automatic magnitude processing of positive numerical magnitudes. The shift of spatial attention is more feasible when the magnitudes are presented in the positive number format as numerical magnitudes are the same as absolute values of the numbers (for example, |1|= 1). However, this is not true for the negative number case. For the negative number, a large numerical magnitude (− 1) is a small number in an absolute number sense (for example, |− 1| <|− 9|, although − 1 is to the right of − 9 on the real number line). Therefore, it may be possible that the shift of attention evoked by numerical magnitude may not be so automatic in the case of negative numbers. Hence, it may be the reason why we did not observe the influence of numerical magnitude on temporal processing in the negative number case when presented in a separate block (Experiment-1). Further, we speculate that a similar mechanism might be operating for positive and negative numbers when presented together in an intermixed order (Experiment-2). The polarity of numbers may cause an automatic shift in spatial attention and may lead to differential temporal processing for positive and negative numbers. The arguments invoking the influence of numbers on shifts of spatial attention need to be made with caution considering the recent failure of replication in a multilab study^45^, but also see^46^.

Further, a closer look at the data reveals an interesting pattern that the positive numbers behaved differently across the two experiments. A large positive numerical magnitude is perceived longer than that of a small numerical magnitude when presented in a separate block (experiment-1, Fig. 2). However, the influence of positive numerical magnitude on temporal processing disappears when positive numbers were presented with negative numbers in an intermixed order as in experiment-2 (see Fig. 4). One can ask why positive numbers behave differently across the different experimental conditions. We speculate that the anchor (reference) magnitude plays a key role in setting up the context. For example, in experiment-1, the positive numbers “1” and “9” were presented with a reference number “5”. Whereas in experiment-2, the positive (as well as negative) numbers were presented against a common reference “0”. Although the numerical distance (between 1 and 9) is sufficiently large to provide a sense of relative numerical magnitude (small and large) in both the experiments, we did not observe any difference in temporal processing for 1 and 9 in experiment-2, indicating a possible context effect. This suggests that the polarity context drives differential temporal processing for the same absolute numbers. On the other hand, negative numbers seem to behave similarly across the two experiments in that the relative magnitude does not seem to influence temporal processing in both the experiments.

To conclude, the present study investigated the influence of task-irrelevant numerical magnitudes on duration judgments using positive and negative numbers in two different settings (experiments). The findings suggest that positive numbers (1 versus 9) affect duration judgments. On the other hand, the negative numbers did not interfere with duration judgments when presented in a separate block. Further, the magnitude effect seen for positive numbers (1 versus 9) seems to disappear when positive and negative numbers were presented in an intermixed order. The findings also provide evidence that the processing of the polarity of numbers affects duration judgments but not the number magnitude. Our results also indicate that the number-time interaction may be mediated by the spatial relations of the two magnitudes. This is particularly evident when the two magnitudes were presented in such a way that we could represent them in a spatial format (for example, 1 versus 9 in the context of 5 in experiment-1, − 1 versus 1 in the context of 0 in experiment-2, etc.), then number interacted with temporal processing. However, when the two magnitudes were presented in such a way that no spatial representation was possible or can be formed (for example, − 1 versus − 9 in the context of − 5 in experiment-1, 1 versus 9 in the context of 0 in experiment-2, etc.), we did not observe the numerical influence on temporal processing. Thus, we suggest that the number-time interaction arises from the modulation of attentional mechanisms and may not be processed by the generalized magnitude system proposed by Walsh^1^. In the future, we should conduct experiments where the numbers are presented in the left and right of space instead of presenting them in the center to validate the alternative explanation proposed here.

## Methods

### Experiment-1: positive and negative number magnitudes (block design)

#### Apparatus

The stimuli were presented and controlled using E-Prime^47^ Standard-2.0 on a 17-inch Samsung CRT monitor (1024 × 768 resolution) running at a frame rate of 100 Hz.

#### Participants

A total of 35 right-handed naïve participants (19 male) were recruited from the International Institute of Information Technology, Hyderabad, India. The age range of the participants was 20–28 years. They had normal or corrected-to-normal vision. All the experimental procedures and methods were performed in accordance with the relevant guidelines and regulations and the study was approved by the Institute Review Board (IRB), International Institute of Information Technology, Hyderabad, India. Informed consent forms were obtained from all the participants, and remuneration was paid for their participation.

#### Stimulus and procedure

Participants were taken to a dimly lit experimental room. They were asked to sit comfortably. The distance between the participant and the monitor was 57 cm. A temporal comparison task was used, and the participants were asked to discriminate whether the comparison stimulus lasted for a longer duration compared to the standard stimulus. Each participant performed two number blocks: *Positive* and *Negative* blocks. In the positive number blocks, the numerals “1” and “9” were used as comparison stimuli and the numeral “5” as a standard stimulus.

Similarly, for the negative number blocks, the numbers “ − 1” and “ − 9” were used as comparison stimuli and the number “ − 5” as the standard stimulus. The motivation behind using two different (positive and negative) sets of numbers is to have control over the absolute number at the same time *negative* sign makes the magnitude different for the same absolute number. For example, in the positive set, the number “1” represented a small numerical magnitude, whereas “9” represented a large numerical magnitude. On the contrary, the negative number “ − 1” was used to represent a large numerical magnitude, and “ − 9” represented a small numerical magnitude. The polarity of the number (positive and negative) was blocked, and the order of the presentation was counterbalanced across the participants.

All the stimuli were presented at the center of the screen and were of a 2° visual angle. Each trial started with a self-paced fixation cross, followed by the standard stimulus (“5” in positive and “ − 5” in the negative block) for a fixed duration of 500 ms. The second number (comparison stimulus) was presented for nine varied durations from 100 to 900 ms in steps of 100 ms. The comparison and the standard stimuli were separated by an inter-stimulus-interval (ISI) of 1000 ms. At the end of the trial, a white blank screen was presented until a response was received. Participants were instructed to judge whether the second stimulus lasted longer or shorter compared to the standard stimulus. They were also asked to make their judgments independent of the presented numbers. Participants executed their responses by pressing a dedicated key on the keyboard for the long and the short responses. The long and short response key-mapping was counterbalanced across the subjects. Each participant performed 144 trials of each of the positive and negative blocks. Each block contained 2 (Magnitude: Small and Large) × 9 (Durations: 100–900 ms in steps of 100 ms), and each duration was repeated 8 times. The stimulus presentation and the block order were randomized across the subjects. After the experiment, participants were given a magnitude judgment task to make sure that they knew which magnitude was larger and smaller, especially for the negative numbers. All the participants correctly judged the large magnitude value in negative as well as positive numbers.

### Experiment-2: positive and negative number magnitudes (intermixed design)

#### Participants

A total of 31 participants (21 male) were recruited from the International Institute of Information Technology, Hyderabad, India. The age range of the participants was 23–28 years. All the participants reported normal vision. Informed consent forms were obtained from all the participants and remuneration was paid for their participation.

#### Stimulus and procedure

All the stimulus and procedure were identical to experiment-1 except that the positive and negative numerical magnitudes were presented in an intermixed order. Unlike in experiment-1, here, we presented “0” as the standard stimulus. Apart from these two changes, the protocol of experiment-2 was identical to that of experiment-1. Similar to experiment-1, each participant performed a total of 288 trials. The trials comprised 4 (Numbers: − 9, − 1, 1, 9) × 9 (Durations: 100–900 ms in steps of 100 ms), and each duration was repeated 8 times. Like in experiment-1, participants were given a magnitude judgment task after the experiment to make sure that they knew which magnitude was larger and smaller, especially for the negative numbers. All the participants correctly judged the large magnitude value in negative as well as positive numbers.

## Supplementary Information

Supplementary Information.
