# Supplementary material for: Attention mediates the influence of numerical magnitude on temporal processing
Source: Sci Rep. 2021 May 26;11:11030. doi: 10.1038/s41598-021-90466-2 (PMC8155092; doi:10.1038/s41598-021-90466-2)
Supplement: Supplementary file 1 — Supplementary Information. [file 41598_2021_90466_MOESM1_ESM.pdf]

# Attention Mediates the Influence of Numerical Magnitude on Temporal Processing

Anuj Shukla & Raju S Bapi*

*Cognitive Science Lab, International Institute of Information Technology, Kohli Research Block, Gachibowli, Hyderabad, 500032, Telangana, India*

***Corresponding Author email: raju.bapi@iiit.ac.in**

# Supplementary Materials

## Experiment-1: Positive and Negative Numbers (Blocked)

In addition to the analysis presented in the main text, we also analysed the magnitude of the null result observed for the negative number block in experiment-1. To check whether a large numerical magnitude is perceived to last longer than the small numerical magnitude within negative number blocks. The Bayesian paired sample t-test was conducted using JASP. For the negative number block, the estimated Bayes factor (null/alternative) suggested that the data were 11.54 times more likely to occur under the null hypothesis than under the alternative hypothesis. The estimated Bayes factor ($B_{10}$=11.54) provides substantially strong evidence for the null hypothesis suggesting that the numerical magnitude does not affect temporal perception in the case of a negative number block.

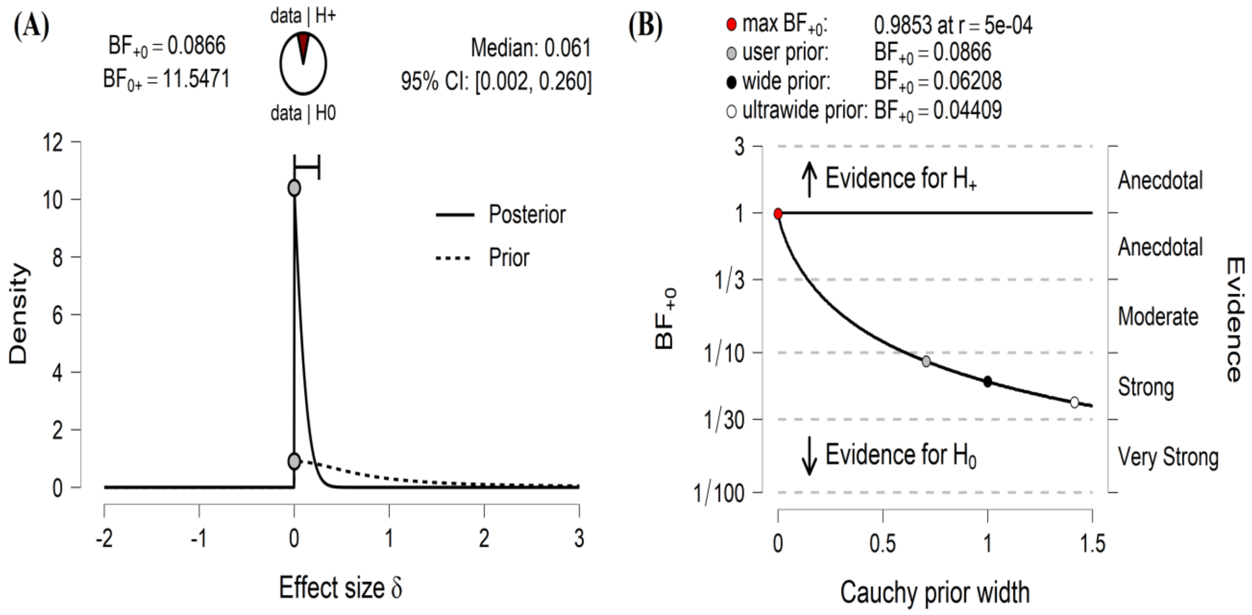


**Supplementary Figure-S1**: Bayesian analysis of the mean PSE differences for negative numbers (A) The prior and posterior distribution plot (B) A robustness check showing the effects of assigning wide and ultrawide Cauchy prior widths on Bayes factor values.

**Experiment-2: Positive and Negative Numbers (Intermixed)**

In addition to the main analysis presented in the article, we also performed a Bayesian paired sample t-test to estimate the magnitude of the null results obtained while comparing the numerical magnitude within positive and negative number domains. In a positive number domain (1 vs 9), the Bayes factor analysis suggested that the data is 3.29 times more likely to occur under the null hypothesis than the alternate hypothesis. The Bayes factor of 3.29 demonstrates moderate evidence in favor of the null hypothesis.

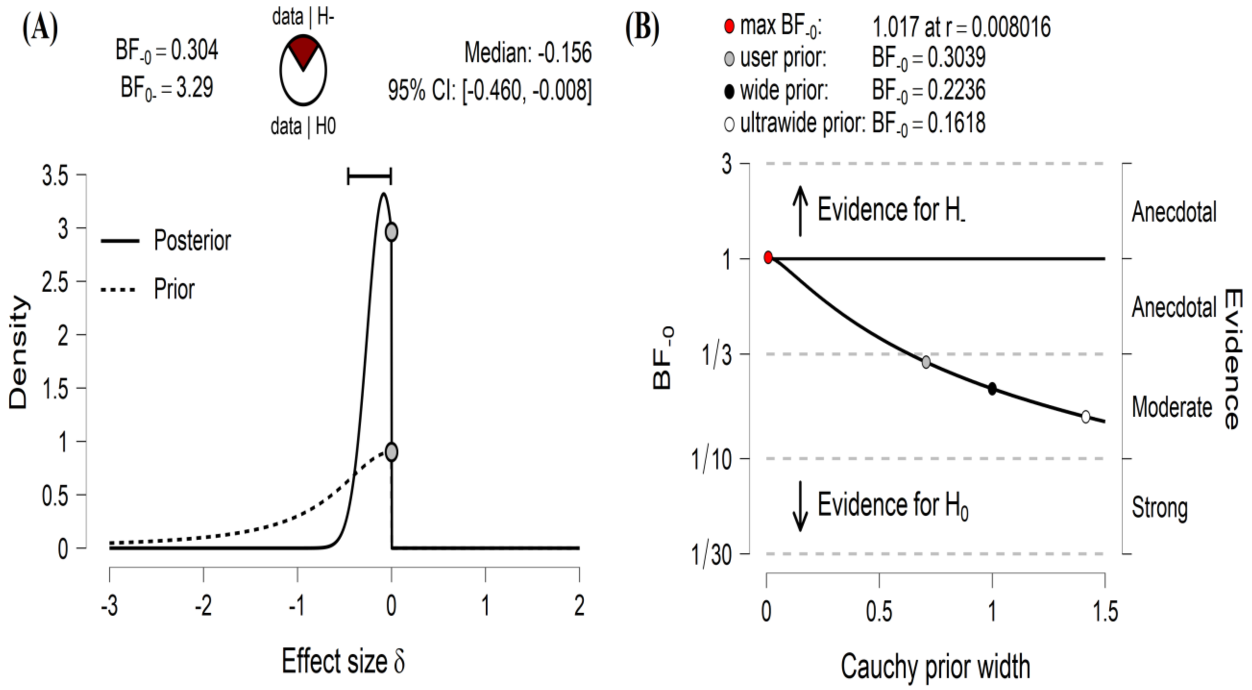


**Supplementary Figure-S2**: Bayesian analysis of the mean PSE differences for positive number domain (A) The prior and posterior distribution plot (B) A robustness check showing the effects of assigning wide and ultrawide Cauchy prior widths on Bayes factor values.

Similarly, the results of the negative number domain (-1 vs -9) indicated that the data is 7.195 times more likely to occur in the null hypothesis compared to the alternate hypothesis. The Bayes factor of 7.195 suggestive of moderate to strong evidence in favor of the null hypothesis. Taken

together, the overall Bayesian analysis suggests that the numerical magnitude does not modulate temporal processing when compared within the positive and negative number domain.

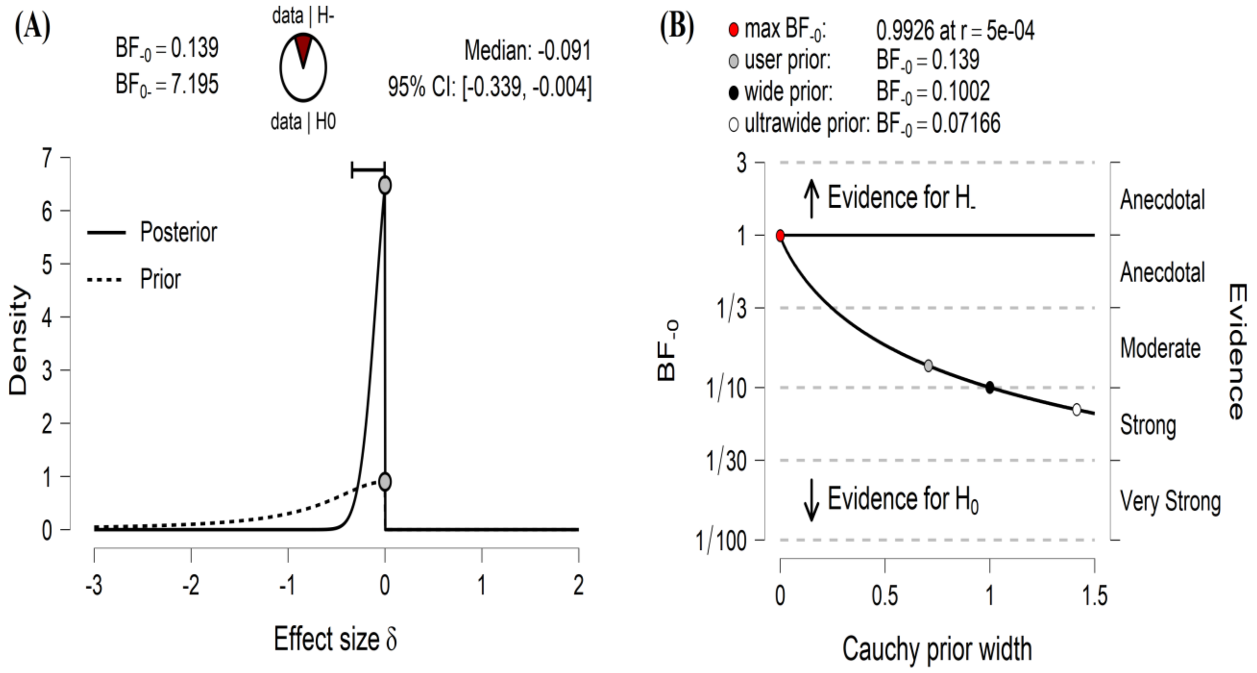


**Supplementary Figure-S3**: Bayesian analysis of the mean PSE differences for negative number domain (A) The prior and posterior distribution plot (B) A robustness check showing the effects of assigning wide and ultrawide Cauchy prior widths on Bayes factor values.

**Average Psychometric Plot for Experiment-1: Positive and Negative Numbers (Blocked)**

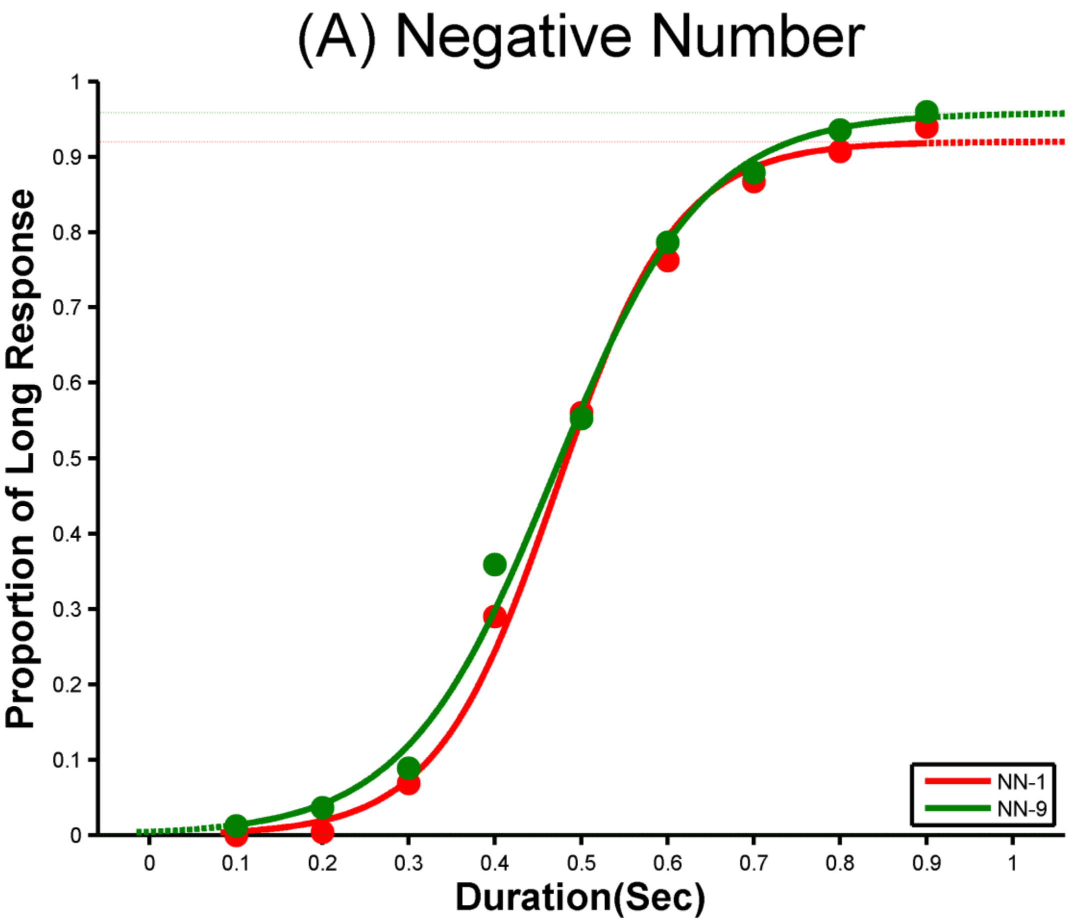


**Supplementary Figure-S4 (A)**: shows an average psychometric plot for the negative block (Experiment-1). The large numerical magnitude “-1” (NN-1) is indicated in red, and the small numerical magnitude “-9” (NN-9) is in green.

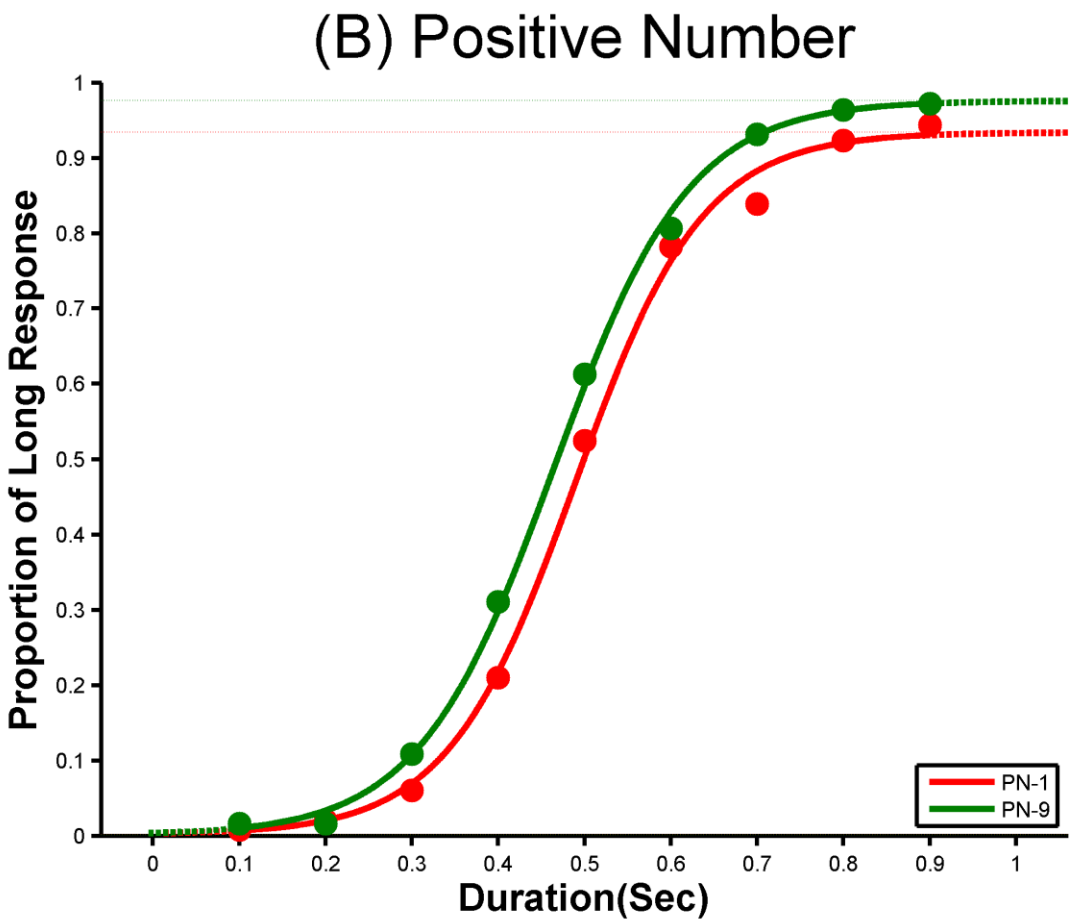


**Supplementary Figure-S4 (B)**: shows an average psychometric plot for positive number block (Experiment-1). The large numerical magnitude “9” (PN-9) is indicated in green, and the small numerical magnitude “1” (PN-1) is in red.

## Average Psychometric Plot for Experiment-2: Positive and Negative Numbers (Intermixed)

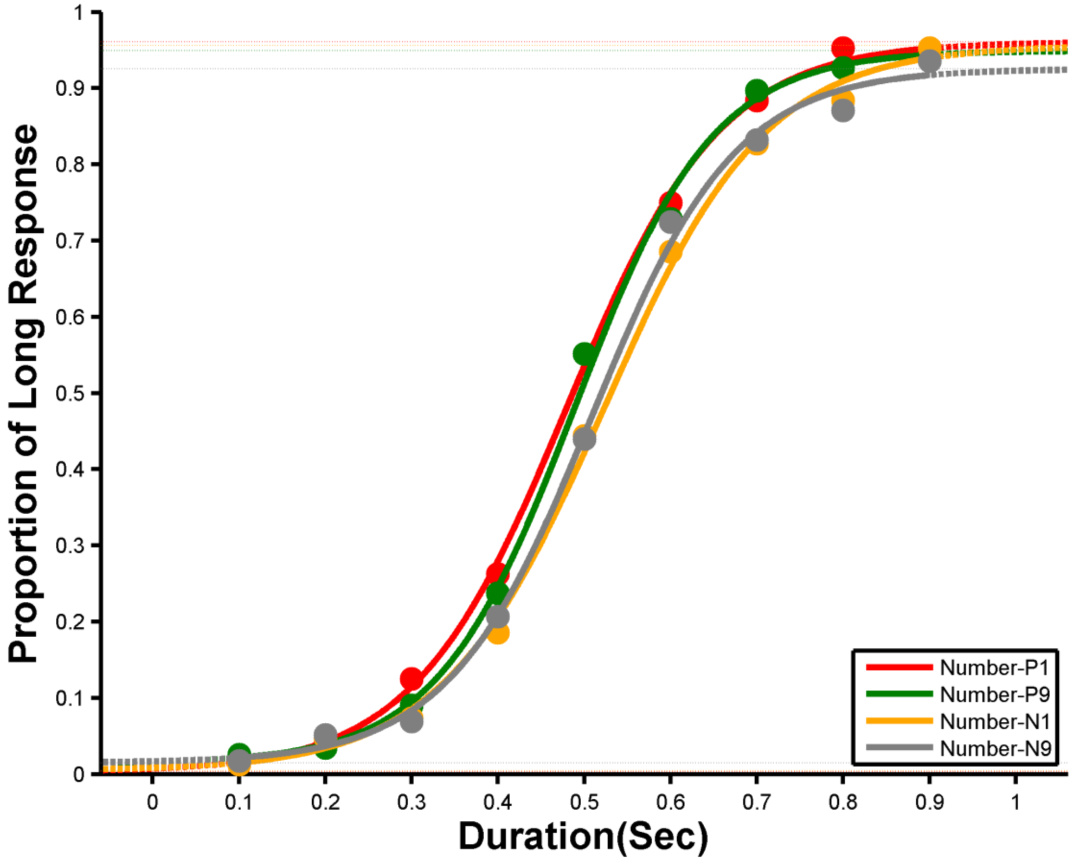


**Supplementary Figure-S5**: shows an average psychometric plot for positive and negative numbers (Experiment-2). The large positive numerical magnitude "9" (Number-P9) is indicated in green and the small positive numerical magnitude "1" (Number-P1) is in red. The large negative numerical magnitude "-1" (Number-N1) is shown in yellow and the small negative numerical magnitude "-9" is in gray.
